# Prion Amyloid Polymorphs – The Tag Might Change It All

**DOI:** 10.3389/fmolb.2020.00190

**Published:** 2020-08-06

**Authors:** Luc Bousset, Nina Luckgei, Mehdi Kabani, Carole Gardiennet, Anne K. Schütz, Ronald Melki, Beat H. Meier, Anja Böckmann

**Affiliations:** ^1^Institut Francois Jacob (MIRCen), CEA and Laboratory of Neurodegenerative Diseases, CNRS, Fontenay-aux-Roses, France; ^2^Molecular Microbiology and Structural Biochemistry, UMR 5086 CNRS, Universite de Lyon, Lyon, France; ^3^ETH Zürich, Laboratory of Physical Chemistry, Zurich, Switzerland

**Keywords:** prion, fibrils, Sup35pNM, NMR, affinity tags, polymorphs

## Abstract

Sup35p is a protein from *Saccharomyces cerevisiae*. It can propagate using a prion-like mechanism, which means that it can recruit non-prion soluble Sup35p into insoluble fibrils. Sup35p is a large protein showing three distinct domains, N, M and an extended globular domain. We have previously studied the conformations of the full-length and truncated NM versions carrying poly-histidine tags on the N-terminus. Comparison with structural data from C-terminally poly-histidine tagged NM from the literature surprisingly revealed discrepancies. Here we investigated fibrils from the untagged, as well as a C-terminally poly-histidine tagged NM construct, using solid-state NMR. We find that the conformation of untagged NM is very close to the N-terminally tagged NM and confirms our previous findings. The C-terminal poly-histidine tag, in contrast, drastically changes the NM fibril structure, and yields data consistent with results obtained previously on this construct. We conclude that the C-terminally located Sup35p globular domain influences the structure of the fibrillar core at the N domain, as previously shown. We further conclude, based on the present data, that small tags on NM C-terminus have a substantial, despite different, impact. Modifications at this remote localization thus shows an unexpected influence on the fibril structure, and importantly also its propensity to induce *[PSI+]*.

## Introduction

The Sup35p protein is a prion from *Saccharomyces cerevisiae. [PSI+]*, its loss-of-function phenotype, appears after spontaneous assembly of Sup35p and causes a tRNA-mediated suppression of nonsense mutations (Cox, 1965). *[PSI*+*]* was first discovered by genetic screens (Cox, 1965). It can propagate *via* a prion-like mechanism. In contrast to the human prion protein, PrP, whose cellular function is not fully understood, Sup35p is known to play a crucial role in translation termination. *[PSI+]-*mediated nonsense suppression can result in the synthesis of a functional polypeptide even though a stop-codon has arisen through mutation in an open reading frame, thereby suppressing the mutant phenotype. Sup35p is a GTP-binding protein that interacts with Sup45p to form the release factor that recognizes an in-frame stop codon in an mRNA (Stansfield et al., 1995; Zhouravleva et al., 1995). In the context of *[PSI+]*, Sup35p is associated within stable high-molecular-weight aggregates, which reduces the efficiency of termination at stop codons. This results in read-through stop codons in up to 20% of the time.

Sup35p counts 685 amino-acid residues and contains three domains (Ter-Avanesyan et al., 1993). The N-terminal domain, including residues 1–123, is necessary and sufficient for *[PSI+]* occurrence and maintenance. Deletion of this domain allows Sup35p to remain soluble even in [*PSI*+] cells (Paushkin et al., 1996). The M-domain, including residues 124–253, is highly charged and plays a role in the solubility of non-prion Sup35p and modulates [*PSI*+] propagation (Liu et al., 2002). The C-terminal domain houses the translation termination activity (Zhouravleva et al., 1995) and retains a native and active conformation, even in the context of Sup35p fibrils (Krzewska et al., 2007; Luckgei et al., 2013).

The constructs investigated in this study are the truncated versions Sup35pNM (amino acids 1–253), devoid of, or carrying poly-histidine tags. Indeed, the prion properties of Sup35pNM have been extensively documented by various methods of structural and functional biology using versions of the protein bearing N- and C-terminal poly-histidine tags (Glover et al., 1997; Tanaka et al., 2004; Krishnan and Lindquist, 2005; Shewmaker et al., 2006, 2009; Krzewska et al., 2007; Toyama et al., 2007; Shorter and Lindquist, 2008). Until now, the influence of the N- or C-terminal poly-histidine tag on the structure of the Sup35pNM fibrils was not investigated at molecular level. We have previously shown that there are significant structural differences between N-terminal poly-histidine tagged full length/authentic Sup35p and Sup35pNM, even if the fibrillar core in both fibrils was found to localize to the first 30 N-terminal residues (Luckgei et al., 2013, 2014; Schütz et al., 2014). Those differences may be due to the C-terminal, compactly folded Sup35p moiety. However, since the assigned residues of the fibril’s core are located at the very N-terminus of Sup35p, almost immediately after the N-terminal poly-histidine tag, we decided to evaluate the impact on the structural features of the fibrillar form of the Sup35pNM protein of the two poly-histidine tags MGSSHHHHHHSSGLVPRGSH (on the N-terminus, His-Sup35pNM) or GSHHHHHH (on the C terminus, Sup35pNM-His). This investigation was further motivated by the observation of a strong Met signal, as well as several broad serine signals in the spectra of N-terminally poly-histidine tagged Sup35pNM that could indeed stem from the poly-histidine tag itself (Luckgei et al., 2013, 2014; Schütz et al., 2014). We show here that when it is present on the C-terminal end, the poly-histidine tag significantly affects the folding landscape of Sup35pNM-His, yielding different fibrillar polymorphs. At the same time, we observe that its N-terminal localization does not significantly alter the structure of His-Sup35pNM when compared to untagged Sup35pNM, validating our previous investigations of N-terminally poly-histidine tagged Sup35p.

## Materials and Methods

### Expression, Labeling, Purification and Assembly of Sup35pNM

The Sup35p and Sup35pNM N and C-terminally tagged proteins were overexpressed and ^15^N, ^13^C labeled as described previously (Luckgei et al., 2013) in *Escherichia coli* strain BL21-CodonPlus and purified by affinity chromatography as previously described (Krzewska and Melki, 2006; Krzewska et al., 2007). Assembly reactions were performed at a final concentration of 8–16 μM in assembly buffer (50 mM Tris–HCl, pH 8.0, 200 mM NaCl, 5% glycerol, 5 mM beta-mercaptoethanol, 10 mM MgCl_2_, 2 mM EGTA) for 30 days at 6°C under very mild agitation (<100 rpm) as described previously (Krzewska and Melki, 2006; Kabani et al., 2011).

### Yeast Strains, Growth Media and Monitoring of Prion Phenotypes

The *S. cerevisiae* strain used in this study is 74-D694 [MATa ade1-14 (UGA) trp1-289 leu2-3,112 his3Δ-200 ura3-52]. Yeast cells were grown in YPDA medium (1% yeast extract, 2% peptones, 2% glucose, 0.002% adenine). Prion phenotypes were monitored on 1/4-YPD medium (0.25% yeast extract, 2% peptones, 2% glucose, 2% bacto-agar) via a standard color-based phenotype assay, as described before (Kabani et al., 2011, 2014).

### Induction of [PSI^+^] by Transformation of Yeast Spheroplasts

Yeast 74-D694 [*psi*^–^] cells were converted to spheroplasts by lyticase treatment and then transformed with His-Sup35pNM or Sup35pNM-His fibrils (5 μg) in the presence of 100 μg ml^–1^ of salmon sperm DNA and 20 μg ml^–1^ of the URA3-based pRS316 plasmid, as described before (Kabani et al., 2011, 2014). Just before transformation, *in vitro* assembled fibrils were briefly sonicated on ice 4 times for 5 s with 20 s pauses between each sonication using a Misonix S-4000 sonicator with a microtip at 20% amplitude. Mock transformation reactions lacking spheroplasts and/or pRS316 ensured that no viable cells remained in the cell extracts and the absence of spontaneous [*ura*^+^] revertants.

### Semi-Denaturant Detergent Agarose Gel Electrophoresis (SDD-AGE)

Yeast cells (∼20 OD_600nm_ units) were harvested by centrifugation at 4,000 *g* for 2 min and resuspended in 500 μl of lysis buffer (100 mM Tris-Cl pH 7.5, 50 mM KCl, 10 mM β-mercaptoethanol, 0.5% Triton X-100, 1 mM PMSF and protease inhibitor cocktail, Roche Diagnostics). Glass beads were added to half the cell suspension volume and cells were broken by six cycles of vortexing for 30 s with 1 min incubation on ice between each vortexing. Debris and unbroken cells were removed by centrifugation for 2 min at 4,000 *g* and at 4°C. SDD-AGE analysis was performed as described previously (Wang et al., 2017). Immunoblots were developed using enhanced chemiluminescence reagents (Pierce) and a Chemidoc imaging system (Biorad).

### NMR Spectra

Solid-state NMR spectra were obtained from uniformly ^13^C,^15^N-labeled protein pelleted at 100,000 g and ultracentrifuged into the NMR rotor (Böckmann et al., 2009). NMR experiments were performed as described in Luckgei et al. (2014). Briefly, they were recorded on Bruker Avance II spectrometers operating at 800 and 850 MHz field strengths equipped with 3.2 mm triple-resonance MAS probe at 17.5 kHz MAS spinning frequencies and a sample temperature around 5°C. DARR (Takegoshi et al., 2003), NCA (Baldus et al., 1998), NCACB (Schuetz et al., 2010; Habenstein et al., 2011), ^1^H-^13^C INEPT (Siemer et al., 2006), and T_1_-filtered ^13^C -^13^C correlation spectra (Luckgei et al., 2013) were recorded on protein samples filled in the 3.2 mm rotors using ultracentrifugation (Böckmann et al., 2009).

## Results

**FIGURE 1:**
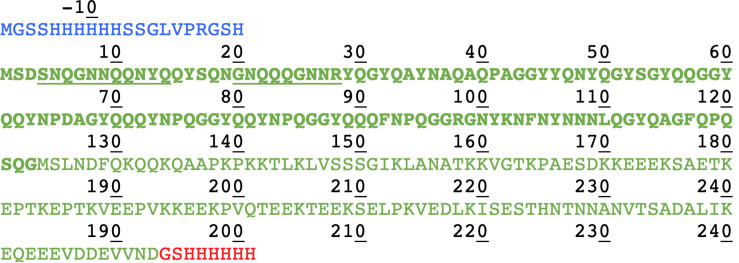
Primary structure of Sup35pNM with an N- and C-terminal poly-histidine tag. The N-terminal poly-histidine tag is colored in blue, the C-terminal one in red. The assigned residues (Luckgei et al., 2013, 2014) are underlined.

We compared in this work the solid-state NMR spectra of the fibrillar forms of His-Sup35pNM, Sup35pNM-His and untagged Sup35pNM. In the primary structure shown in Figure 1, the sequences of Sup35pNM (green, N in bold), the N-terminal poly-histidine tag (blue) and the C-terminal poly-histidine tag (red) are outlined. The previously assigned amino acids, comprising residues 4–14 and 20–28 (Luckgei et al., 2013, 2014), are underlined.

### N-Terminally Tagged and Untagged NM Show Very Similar Conformations

**FIGURE 2:**
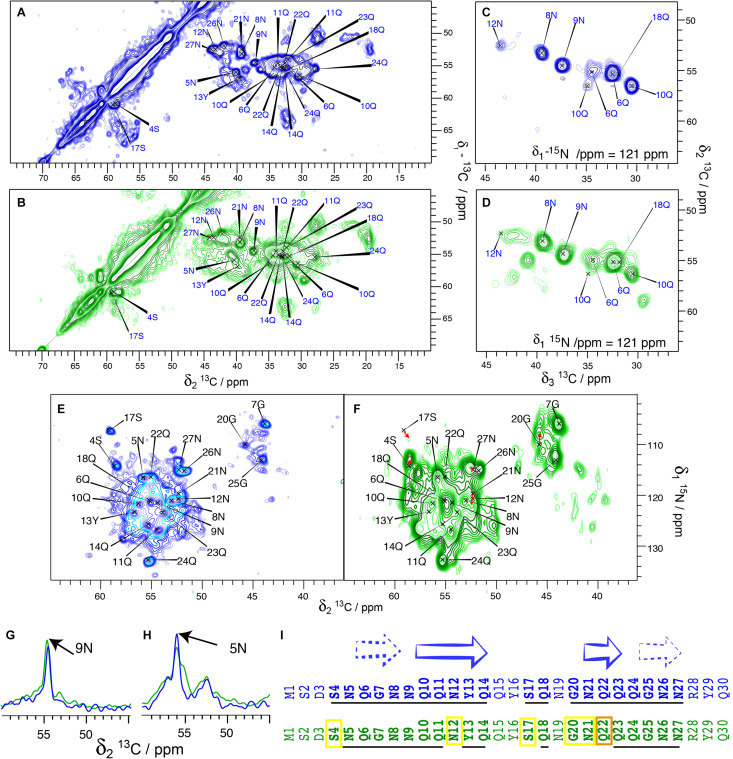
Comparison between His-Sup35pNM (blue) and untagged Sup35pNM (green). All spectra from His-Sup35pNM (blue) are from references (Luckgei et al., 2013, 2014), including assignments of the fibril core of His-Sup35pNM are shown as previously established there. **(A)** 20 ms ^13^C-^13^C DARR correlation spectra of His-Sup35pNM and **(B)** untagged Sup35pNM, as well as representative planes of 3D NCACB spectra of **(C)** His-Sup35pNM and **(D)** untagged Sup35pNM. Panels **(A,C)** were recorded at 850 MHz (Luckgei et al., 2013, 2014); Panels **(B,D)** were recorded at 800MHz proton frequency; all with a MAS spinning frequency of 17.5 kHz and 5°C sample temperature. **(E)** NCA spectrum of His-Sup35pNM and **(F)** untagged Sup35pNM. The spectrum in panel **(F)** was recorded at 850 MHz proton frequency (12 ms acquisition in F2, 8 scans) (Luckgei et al., 2013, 2014) and in panel **(E)** at 800 MHz proton frequency (6 ms acquisition in F2, 16 scans); both spectra were measured at a MAS spinning frequency of 17.5 kHz and 7°C sample temperature. Red arrows suggest possible chemical-shift changes from His-Sup35pNM to untagged Sup35pNM. **(G,H)** 1D traces from 20 ms DARR spectra of His-Sup35pNM (blue) and untagged Sup35pNM (green). The spectra were recorded 850 MHz proton frequency (His-Sup35pNM) (Luckgei et al., 2013, 2014) and 800 MHz proton frequency (untagged Sup35pNM) at 7°C sample temperature and with a MAS spinning frequency of 17.5 kHz. The assignments of 9N and 5N are indicated. **(I)** Comparison of His-Sup35pNM (blue) and untagged Sup35pNM (green). Secondary structures derived from secondary chemical shifts of the assigned residues from the Sup35pNM-Ntag fibril core are shown according to Luckgei et al. (2013). The assigned residues in the His-Sup35pNM spectra and residues which are visible in the untagged Sup35pNM spectra at the same chemical shift are underlined. Yellow boxes indicate which residues are visible in untagged Sup35pNM with a slight chemical shift change, and in orange the residue which could not be traced.

We have, in our previous studies (Luckgei et al., 2013, 2014), used His-Sup35pNM. In order to assess whether the tag changes the structural features of the protein, we performed a comparison between untagged and His-Sup35pNM using 2D DARR and 3D NCACB solid-state NMR spectra. An analysis of 20 ms DARR, NCA and NCACB fingerprint spectra reveals that untagged Sup35pNM is indeed very similar to the previously studied His-Sup35pNM with respect to line positions in the NMR fingerprints indicating very similar structures (Figures 2A,B), as well as to signal-to-noise ratio (and thus dynamic behavior). Most of the assigned residues of the Sup35pNM amyloid fibril core have highly similar chemical shifts and comparable peak intensity in the spectra of the two fibrils. 3D NCACB spectra confirm this analysis (Figures 2C,D). Few minor differences can be observed: The serine residue 4S is shifted by 1 ppm in the ^15^N-dimension, but shows identical Cα and Cβ chemical shifts. It can be noted that 4S is located at the beginning of the assigned stretch and is the closest residue to the N-terminal poly-histidine tag. Residue 12N shows a slightly different chemical shift for Cα, Cβ, and N. Further, residue 22Q seems to be shifted in the ^15^N dimension in NCACB spectra.

The 2D NCA spectra of His-Sup35pNM and untagged Sup35pNM are given in Figures 2E,F. The fingerprints of the spectra are very similar, with slightly broader lines for untagged Sup35pNM. Most resonances can be observed at very similar chemical shifts. The resonance frequencies of 4S, 12N, 17S, 20G and 21N might be shifted slightly. Red arrows indicate possible chemical-shift changes, since new unassigned resonances appear close to the prior, now vanishing, resonance frequencies. Figures 2G,H show 1D traces from 20 ms DARR spectra of His-Sup35pNM (blue) and untagged Sup35pNM (green). The traces are representative for many peaks that show a highly similar signal intensity for most of the assigned peaks in both spectra.

The broad serine signals visible in the DARR spectra around 65/57 ppm of the His- Sup35pNM, which likely belong to residues outside the fibrillar core, are weaker in the spectra of the untagged Sup35pNM. This indicates that these residues are probably more rigid in His-Sup35pNM. Conversely, some peaks in untagged Sup35pNM fibrils that are not observed in His-Sup35pNM can be identified in the 20 ms DARR and NCACB (DREAM) spectra indicating that those are rather more rigid. The signals cannot be assigned unambiguously; however, one signal might belong to residue 17S, which has been assigned in the fibril core and disappears in untagged Sup35pNM. The others might be assigned to D and R/K residues. Since only very few new peaks appear, it cannot be known from which domain they might stem and therefore they were not further investigated.

Figure 2I summarizes the resonances, which are observed either at the same chemical shift (underlined) or very likely at an only slightly changed chemical shift (yellow frame) in His-tagged (blue sequence) and untagged Sup35pNM (green sequence). Surprisingly, several chemical-shift changes occurred within predicted β-strands. Especially residue 22Q seems to have shifted more strongly, or disappeared (orange frame). This residue represents the center of a 3-residues β-strand. However, depending on their role for the structural elements (as for example inside/outside of a steric zipper) the residues might be more or less stabilized.

Taken together, our results show that His-Sup35pNM and untagged Sup35pNM fibril cores are highly similar. This similarity includes the observed residues, their chemical shifts and their signal intensities, which also indicates that both fibril cores have a similar dynamic behavior.

**FIGURE 3:**
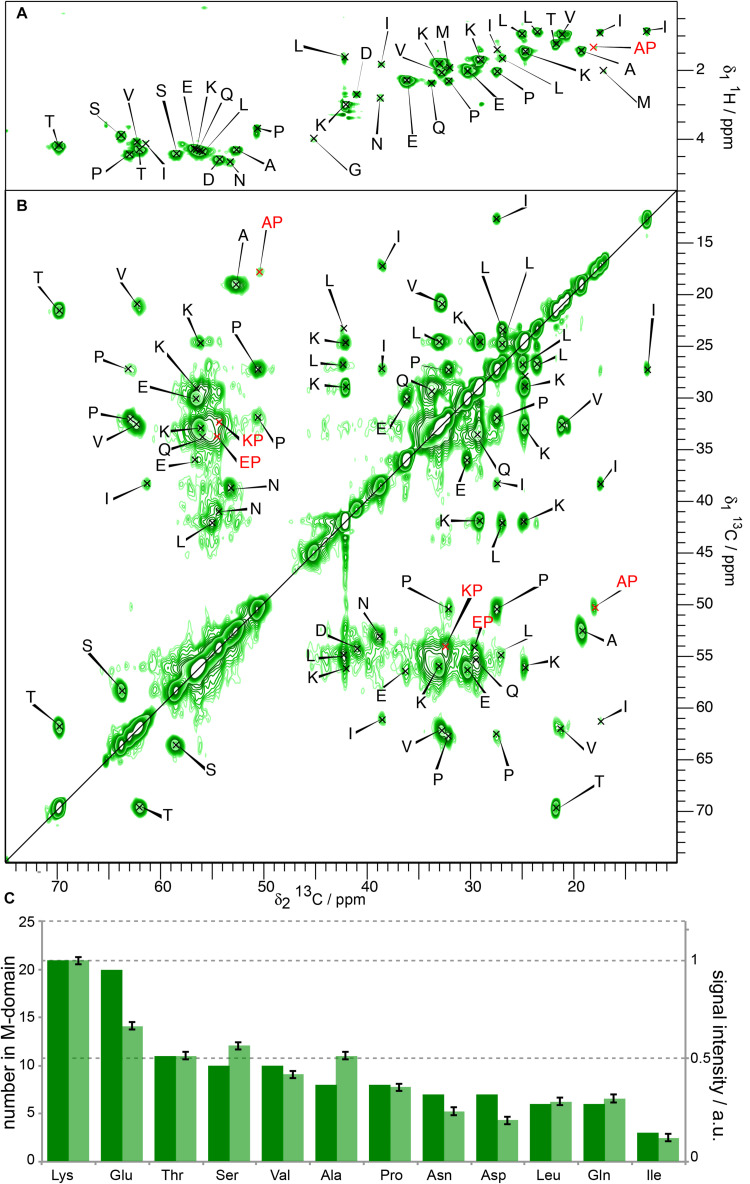
**(A)** ^1^H-^13^C INEPT and **(B)** T_1_-filtered ^13^C-^13^C correlation spectrum (Luckgei et al., 2013) of untagged Sup35pNM. The spectra were recorded at 800 MHz proton frequency and a MAS spinning frequency of 17.5 kHz. The ^13^C-^13^C- correlation spectrum was recorded with a recycle delay of 4 s. The assignments of the random-coil chemical shifts assigned in His-Sup35pNM are indicated. **(C)** Comparison between the signal intensity in T_1_-filtered ^13^C-^13^C correlation spectra (light bars) and the number of the respective amino acid in the M-domain (dark bars) of untagged Sup35pNM. The signal intensity of Lys residues was arbitrarily set to 1, and scaled accordingly for the remaining residues. The error bars indicate the standard deviation of the noise level of each spectrum.

To assess the influence of the N-terminal His-tag on the structure of the M-domain of Sup35pNM, we recorded T_1_-filtered, directly pulsed ^13^C 200 ms DARR and INEPT spectra on untagged Sup35pNM. The waiting time between scans was 4 s favoring more dynamic residues with a faster ^13^C-T_1_ relaxation. Reminiscent of His-Sup35pNM (Luckgei et al., 2013), the resonances of the M-domain of untagged Sup35pNM appear in both ^1^H-^13^C INEPT and T_1_-filtered 200 ms DARR spectra (Figures 3A,B). The resonances appear near the random-coil shifts of the respective residues. A comparison of the peak intensities in the T_1_-filtered 200 ms DARR spectra and the numbers of the corresponding residues in the M-domain reveals that the ratio between signal intensity and the number of residues in the M-domain is very similar for untagged Sup35pNM (Figure 3C) and His-Sup35pNM (Luckgei et al., 2013). We conclude that these signals indeed stem from the M domain in both constructs. Overall, this reveals an analogous T_1_-relaxation property for the M-domain in untagged Sup35pNM and His-Sup35pNM fibrils.

### Extensive Polymorphism Is Detected in C-Terminally Tagged Sup35pNM

**FIGURE 4:**
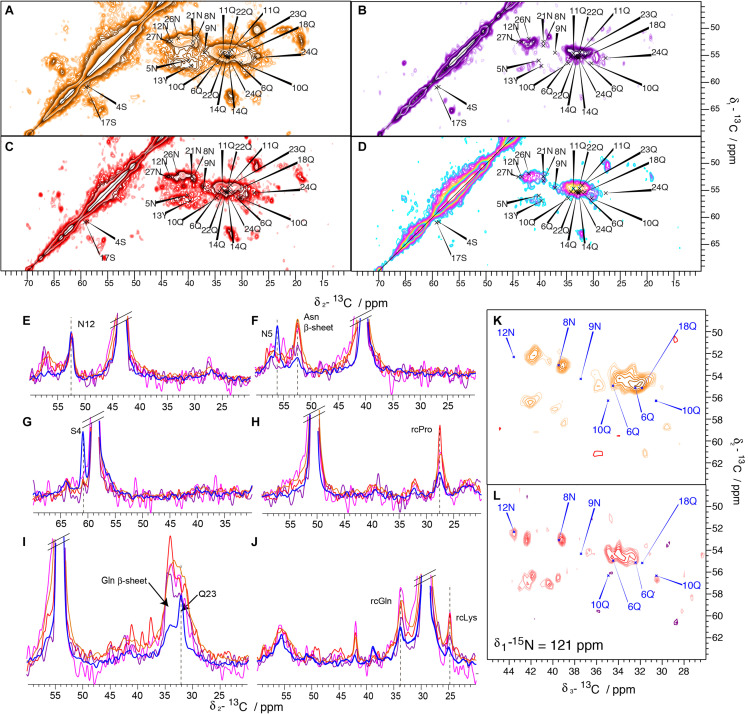
20 ms DARR spectra of His-Sup35pNM and Sup35pNM-His samples. The spectra have been recorded at 850 MHz **(A)** Sup35pNM-His-#1, **(B)** Sup35pNM-His-#2, **(C)** Sup35pNM-His-#3, and 800 MHz **(D)** Sup35pNM-His-#4 proton frequency. The assignments of the fibril core from His-Sup35pNM are indicated in blue. **(E–J)** 1D traces from 2D 20 ms DARR spectra of His-Sup35pNM (blue) and Sup35pNM-His-#1 (orange), Sup35pNM-His-#2 (purple), Sup35pNM-His-#3 (red) and Sup35pNM-His-#4 (pink). The spectra were scaled manually to achieve a similar peak height for 12N. The assignments of some residues from the fibril core, Asn and Gln β-sheet chemical shifts and random coil (rc) chemical shifts are indicated. **(K,L)** Representative planes of 3D NCACB spectra of **(K)** Sup35pNM-His-#1, and **(L)** Sup35pNM- His-#3 samples. The spectra have been recorded at 850, and 800 MHz proton frequency, respectively. The assignments of the fibril core from His-Sup35pNM are indicated in blue.

To analyze the potential effect of a C-terminal His-Tag on the structure of Sup35pNM fibrils, the solid-state NMR spectra of four C-terminally His-tagged Sup35pNM fibrillar preparations, from four independent but supposedly identical Sup35pN-His expressions and purifications, were recorded and compared. The four different spectra exhibited a significant degree of difference, as can be readily observed in 20 ms DARR spectra (Figure 4) from four different samples, prepared following the same protocol and numbered as Sup35pNM-His-#1; (b) Sup35pNM-His-#2; (c) Sup35pNM-His-#3; (d) Sup35pNM-His-#4). Furthermore, Sup35pNM-His-#2 and Sup35pNM-His-#4 exhibited a low signal-to-noise ratio. In contrast to the 20 ms DARR spectrum of His-Sup35pNM, the signals of the fibril core, reported on the spectra, do not contribute to the strongest peaks.

One-dimensional traces from the 20 ms DARR spectra of all four Sup35pNM-His fibrillar samples (orange, purple, red and pink) and previously reported His-Sup35pNM (blue) (Luckgei et al., 2013, 2014) are shown in Figures 4E–J. In order to compare the signal intensity of the assigned peaks, which stem from the fibril core identified in His-Sup35pNM, the spectra were normalized on the 12N resonance that is strong in all spectra (Figure 4E). Many peaks of the assigned residues from the His-Sup35pNM fibrils core seem to disappear in Sup35pNM-His spectra, as for example 4S and 5N (Figures 4F,G). This observation can be due either to dynamical effects (chemical-exchange broadening) or to heterogenous broadening which is difficult to disentangle in the present case. One possibility would be to vary temperature, but it is well known that fibril resonances substantially broaden below the freezing point of water (Bauer et al., 2017), further increasing signal overlap. These effects add to any chemical-exchange broadening, and render temperature-dependent studies of the mechanisms relevant in the physiological temperature range very difficult to interpret.

Since N and Q are the most abundant amino-acid residues within the Sup35p N-domain, which mainly adopts a β-strand conformation, they give rise to strong signals in all spectra. This can be observed in Figure 4F, where a 1D trace not only shows the disappearance of 5N in all Sup35pNM-His fibrils spectra, but also the appearance of a strong new peak at the generic chemical shift of β-strand N residues. A similar phenomenon can be observed in Figure 4I for residue 23Q, which also disappears, and a strong generic chemical shift of β-strand Q residues appearing. Furthermore, Figures 4H,J show that peaks at random-coil resonance positions originating from the M-domain dominate Sup35pNM-His fibril spectra.

Since Sup35pNM-His-#2 and Sup35pNM-His-#4 fibrillar preparations suffered from a lower signal-to-noise-ratio compared to the other samples, despite of the fact that the rotors were filled completely with fibrils, it was not possible to record 3D NCACB spectra in order to investigate these samples in more detail. Sup35pNM-His-#1 and Sup35pNM-His-#3 showed a sufficient signal-to-noise-ratio, so that in each case 3D NCACB spectra could be obtained. The spectra, however, show a poor signal-to-noise ratio compared to His-Sup35pNM. Representative planes of the 3D NCACB spectra for Sup35pNM-His-#1 (Figure 4K) and Sup35pNM-His-#3 (Figure 4L) are shown. Some peaks clearly assigned in His-Sup35pNM spectrum seem to have shifted, some new peaks can be observed and some seem to have disappeared. This indicates that also in 3D NCACB spectra His-Sup35pNM and Sup35pNM-His fingerprints differed.

Altogether, the 2D DARR spectra of Sup35pNM-His fibrils are seemingly dominated by signals corresponding to amino-acid residues in generic random-coil and β-strand conformation but, the latter are not necessarily found the context of a β-sheet (*vide infra*).

**FIGURE 5:**
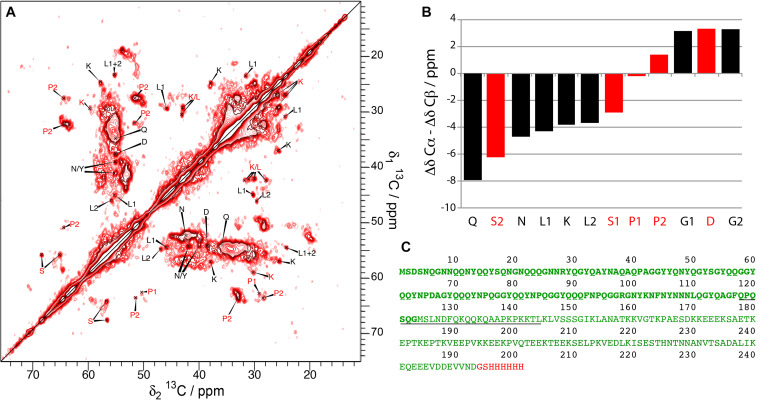
**(A)** 20 ms DARR spectrum of Sup35pNM-His-#3. The spectrum has been recorded at 850 MHz proton frequency, the sample temperature 7°C and the MAS spinning frequency for all spectra 17.5 kHz. The amino-acid specific assignments of the new peaks in the Sup35pNM-His-#3 sample are indicated in red (only visible in 20 ms DARR spectra) and black (visible in 20 ms DARR spectra and 3D NCACB spectra). **(B)** Secondary chemical shifts of the newly assigned peaks in Sup35pNM-His-#3. Black bars: amino acids which could be identified in 20 ms DARR spectra and 3D NCACB spectra; red bars: amino acids which only could be observed in the 20 ms DARR spectra. For glycine, the Cα secondary chemical shift was used. **(C)** Amino-acid sequence of His-Sup35pNM-His, with the C-terminal tag in red. Stretches of amino acids which coincide with the amino acid types that newly appear in the Sup35pNM- His-#3 spectrum are underlined.

The observations we report here clearly suggest different structures and dynamic behavior between different samples related to the absence or the presence of an N- or C-terminal His-tag. While the spectra of Sup35pNM-His-#1, -His-#2 and -His-#4 appear rather featureless, with large blobs at chemical shifts of the amino-acid residues that are most numerous, new strong peaks appear in the spectrum of Sup35pNM-His-#3. These peaks could be assigned tentatively to L, K, P, G, E, S, N, D and H residues considering typical chemical shifts and spin systems (Figure 5A). Although the assignment of the new peaks is only tentative, we derived their secondary chemical shifts to obtain insight into the possible secondary-structure elements which might embed these residues. Most of the residues have secondary chemical shifts typical for a β-strand conformation (Figure 5B). However, it is only when three or more residues in a row show negative secondary chemical shifts that they are indicative of a β-sheet (Wishart and Sykes, 1994).

A possible amino-acid stretch represented in Figure 5B includes residues 118–144 (underlined in Figure 5C), although residues M, F, and A are missing. As the stretches of amino acids between the missing ones are at least 4 amino acids long, they could in principle form three β-strands (Figure 5C).

**FIGURE 6:**
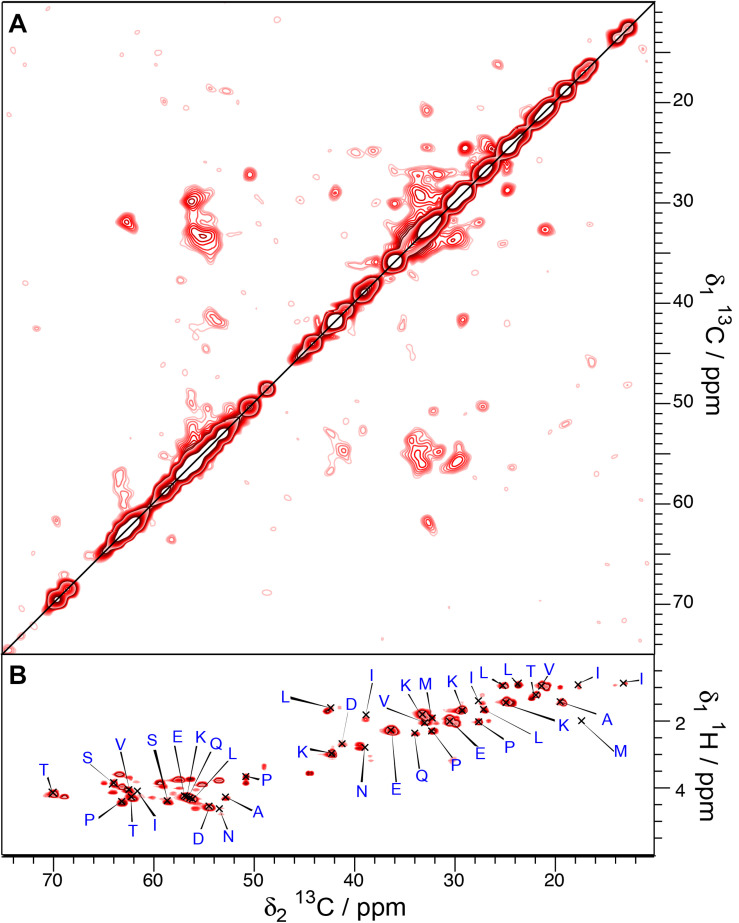
T_1_-filtered ^13^C-^13^C correlation **(A)** and INEPT **(B)** spectra of Sup35pNM-His-#3. The spectra have been recorded at 800 MHz proton frequency, the recycle delay for the T_1_-filtered spectra was 4 s, the sample temperature 7°C and the MAS spinning frequency for all spectra 17.5 kHz.

To probe for the dynamic behavior of Sup35pNM-His-#3, directly pulsed T_1_-filtered DARR and INEPT spectra were recorded (Figures 6A,B). The T_1_-filtered spectra (interscan delay 4 s) show a lower signal-to-noise ratio although they have been recorded 3× as long as for His-Sup35pNM. Since the signals of the T_1_-filtered 100 ms DARR spectra have been assigned exclusively to the M-domain in His-Sup35pNM (Luckgei et al., 2013), this might indicate that the M-domain has a different mobility in Sup35pNM-His-#3. In contrast, several new resonances appear in the INEPT spectra, which cannot be assigned to random-coil chemical shifts. Until now, these peaks remain unexplained.

Altogether, this indicates that in the Sup35pNM-His-#3 sample, parts of the M domain shows different dynamics, with parts of it more rigid, and several residues in β-strand conformation, corresponding either to β-sheet or aggregated regions.

### The Infectious Properties of N-Terminally and C-Terminally His-Tagged Sup35pNM Are Different

**FIGURE 7:**
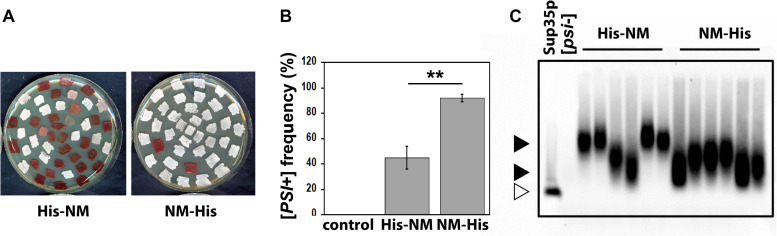
**(A)** 74D-694 [*psi*^–^, *pin*^–^ ] cells were transformed with 5 μg of *in vitro* assembled His-Sup35pNM (His-NM) or Sup35pNM-His (NM-His) fibrils, or with buffer as a control. Random clones from each transformation reaction were then patched on 1/4-YPD plates to assess their prion phenotype (white: [*PSI*^+^]; pink, weak [*PSI*^+^]; red [*psi*^–^ ]). **(B)** Quantification of data such as those presented in panel **(A)**. Error bars represent the standard deviation from three independent experiments, each performed in triplicate (**P < 0.001, unpaired two-tailed Student’s t-test). The [*PSI*^+^] conversion frequency was null when transforming with buffer. **(C)** Cell lysates were prepared from random [*PSI*^+^] clones picked from the plates shown in panel **(A)**, and analyzed by SDD-AGE and immunoblotting using an anti-Sup35p antibody. Large (upper solid arrow head) and small (lower solid arrow head) Sup35p aggregates. Monomeric Sup35p (open arrow head).

Next, we asked whether the different fibrillar structures of His-Sup35pNM and Sup35pNM-His affect their prion properties in vivo. To this end, we used protein transformation assays (Tanaka et al., 2004) to compare the infectious properties of His-Sup35pNM and Sup35pNM-His fibrils (Figure 7). We found that Sup35pNM-His induced [*PSI*^+^] formation with a much higher efficiency than His-Sup35pNM (Figures 7A,B). In agreement with previous data (Tanaka et al., 2004), His-Sup35pNM induced a mixture of strong and weak [*PSI*^+^] clones, as judged by the range of pink to white colors observed on adenine-limiting plates (Figure 7A), and by the differences in the size of SDS-resistant Sup35p aggregates visualized by SDD-AGE (Figure 7C). Strikingly, Sup35pNM-His induced only the formation of strong [*PSI*^+^] clones that were homogenous in color (Figure 7A) and that contained SDS-resistant Sup35p aggregates of comparable sizes (Figure 7C).

## Discussion

Prions have the inherent characteristic to form a variety of variants, or strains, *de novo* or after their re-introduction or propagation into their host cells. This effect seems to be to some extent dependent on the cellular environment and proteostasis. The prion Sup35p yields strong and weak *[PSI+]* phenotypes that are most likely structurally encoded. Sup35_7–13_, and smaller fragments thereof, are highly polymorphic (Sawaya et al., 2007; Lewandowski et al., 2011). We previously demonstrated that even if the fibrillar forms of Sup35p and its NM fragment differ structurally (Luckgei et al., 2013), both show an ordered fibrillar core which is localized within the first 30 residues of the proteins. Thus, although the Sup35pNM domain is critical for *[PSI+]* formation and maintenance (DePace et al., 1998; Toyama et al., 2007), the driving force for fibril formation and the structural characteristics of the fibrils cannot be confined to the NM domain of Sup35p. The results we report here indicate that fibrils made of Sup35pNM bearing a His tag on the C-terminal end of the protein, a construct that has been used in many studies on Sup35pNM (e.g., Glover et al., 1997; DePace and Weissman, 2002; Shorter and Lindquist, 2004, 2008; Tanaka et al., 2005, 2006; Tessier and Lindquist, 2007; Ohhashi et al., 2010; Foo et al., 2011; Verges et al., 2011; Frederick et al., 2015, 2017; Keefer et al., 2017; Shida et al., 2020), and fibrils made of untagged or N-terminally tagged Sup35pNM, are structurally distinct.

Despite the view that fibrils formation is rather driven by the global composition of the prion-forming domain (Ross et al., 2005), it is evident that the very precise interactions that are established between defined amino-acid residues stretches yield the fibril’s amyloid core and determine its structure. Indeed, the side chains of Q and N residues can interdigitate and form a “steric zipper”-like conformations (Nelson et al., 2005), a structure of which has recently been described at high resolution for Orb2 (Hervas et al., 2020). In a more general manner, G and P residues are prone to form turn elements between β-strands, and Y residues might play a role in the initiation of fibril formation through ring stacking. Altogether, the finding that a C-terminal His-tag, at distance from Sup35 N-terminal end, affects the Sup35pNM fibril core structure suggests that changes in the primary structure of the protein even outside the core region can give rise to different fibril core structures.

In addition, major discrepancies are apparent in published structural models for Sup35p fibrils, with models suggesting an overall structural organization in a β-helix (Krishnan and Lindquist, 2005), or supporting parallel in-register β-sheets (Shewmaker et al., 2006). One reason for these differences might be the limited resolution attained so far, that leaves space for individual interpretation of the results. Shewmaker et al. (2006) analyzed selectively Y-, F- and L-labeled fibril samples of C-terminally His-tagged, i.e., Sup35pNM-His, by solid-state NMR spectroscopy. Judging from the relative area underneath the corresponding peaks and the upfield shift compared to the random coil chemical shift in 1D ^13^C spectra, they suggested that 18 out of 20 Y residues, six out of eight L residues, three out of four F and five out of 15 A residues have β-strand conformation. A similar number of residues was determined to have nearest neighbor contacts of about 0.5 nm from which it was concluded that the residues are located in in-register parallel β-sheets. Since about seven out of eight Leu residues were found to have a β-strand conformation and seven L residues are located in the M-domain, it was suggested that the M-domain must have partial β-strand conformation. This data on Sup35pNM-His is to some extent consistent with the data we report here on this construct, since we also see L and K residues with chemical shifts which are typical of β-strand conformation; but they are clearly not consistent with the data we show here on untagged Sup35pNM. The above-mentioned resonances, which can only be observed within Sup35pNM-His, not in His-Sup35pNM nor Sup35pNM, are the strongest new resonances we observe in CP-based spectra. This made it possible to identify several peaks of the same spin system and assign them tentatively to amino-acid types. However, further new resonances appear which are weaker and not assignable. Similar results were obtained by Tessier et al. who also observed an interaction site/recognition element of C-terminal tagged Sup35pNM in the region of residues 90–120 (Tessier and Lindquist, 2007).

We show here that under identical assembly conditions, the context of Sup35pNM, e.g., whether it is within the full-length protein or harboring a short N- or C-terminal tag, influences the resulting final structure of the fibrils. We previously biochemically and functionally documented the influence of the C-terminal domain on Sup35p assembly into fibrils (Krzewska et al., 2007). We also showed that the C-terminal domain affects the overall structure of the fibrils amyloid core (Luckgei et al., 2013). The present results show that as little as a short poly-His tag affects the overall structure of the amyloid form of Sup35pNM domain significantly. Our findings strongly suggest that Sup35pNM conformational landscape is not only affected by the presence of the C-terminal moiety of the protein, but also by smaller additions, even if the C-terminus is distant from the segment forming the amyloid core at the very N-terminal end. Thus, slightly distinct sequences, characteristic of each version of Sup35pNM, can assemble into structurally distinct fibrils.

While the poly-His tag affected marginally the structure of Sup35pNM fibrils (untagged protein) when placed on the N-terminal end of the protein, its positioning at the C-terminal end in contrast radically changed the spectra. Also, C-terminally His-tagged Sup35pNM fibrils displayed a strong degree of polymorphism. Several new resonances could be observed in some samples, which led us to hypothesize that a region lying at the end of the N-domain and the beginning of the M-domain (around residues 120–140) is structured in the presence of a C-terminal His-tag. Studies on two prion variants (Sc4 and Sc37) of C-terminally tagged Sup35pNM (Toyama et al., 2007) revealed H/D exchange protection for both constructs in the region between 110 and 128, in addition to protection of the residues of the fibril core, which lie between residues 1–40. Our results thus support those reports, however, only for C-terminally His-tagged Sup35pNM.

*In vivo* analysis of the functional properties of the different polymorphs we describe yielded clear cut and unexpected results. The polymorphic C-terminally His-tagged NM preparations induced *[PSI+]* formation with a much greater efficiency than their His-Sup35pNM counterparts (Figure 7). Several explanations may account for this observation. As Sup35pNM-His may consist of different polymorphs, it is reasonable to envisage that one or several polymorphs within this mixture have more potent seeding capacity than others, thus, inducing *[PSI+]* with greater efficiency. In addition, the Sup35pNM folding landscape might be more compatible with the structural heterogeneity in Sup35pNM-His, thus, facilitating Sup35pNM molecules recruitment by fibrillar Sup35pNM-His and *[PSI+]* induction. Last, it is not unreasonable to consider that the structural heterogeneity of Sup35pNM-His may yield fibrils with greater probability of being remodeled by breakage or severing in yeast cells. This would yield a larger number of short fibrils and therefore to increased *[PSI+]* induction. The observation that the size of Sup35pNM prion particles induced by the polymorphic Sup35pNM-His is smaller than that induced by His-Sup35pNM (Figure 7C) would be consistent with seeds recruiting endogenous Sup35p with higher efficiency, thus, depleting Sup35p within the cells with greater efficiency and yielding strong *[PSI+]*, as opposed to slowly growing His-Sup35pNM fibrils that yield weak *[PSI+]* and larger prion particles in agreement with the data presented in Figure 7.

Despite significant consistencies between the results we report here and previously published data, caution is required when comparing different datasets, as some lack residue-specific (molecular) resolution. Most importantly, different preparations of C-terminally tagged NM did not yield reproducible spectra. As sample preparation is as tightly controlled as for the other samples, we suspect that this protein construct does not form a single polymorph consistently. Our results clearly show that in addition to a dependence on the experimental conditions, amyloid fibrils polymorphism strongly depends on the context and additions to the amyloid-forming polypeptide chain. Our observations further bring valuable insights into the building principle of structural elements of prion variants.

## Data Availability Statement

The raw data supporting the conclusions of this article will be made available by the authors, without undue reservation.

## Author Contributions

LB prepared all NMR samples. CG, AS, and NL recorded spectra. NL, AS, AB, and BM analyzed NMR data. MK performed protein transformation and SDD-AGE assays. RM, BM, NL, LB, and AB designed study and wrote the manuscript. All authors contributed to the article and approved the submitted version.

## Conflict of Interest

The authors declare that the research was conducted in the absence of any commercial or financial relationships that could be construed as a potential conflict of interest.
